# Supplementary material for: Conserved missense variant pathogenicity and correlated phenotypes across paralogous genes
Source: Genome Biol. 2025 Jul 7;26:197. doi: 10.1186/s13059-025-03663-x (PMC12235991; doi:10.1186/s13059-025-03663-x)
Supplement: Supplementary file 1 — Additional file 1: Contains all supplementary figures S1 to S5. [file 13059_2025_3663_MOESM1_ESM.docx]

**Supplementary Figures**


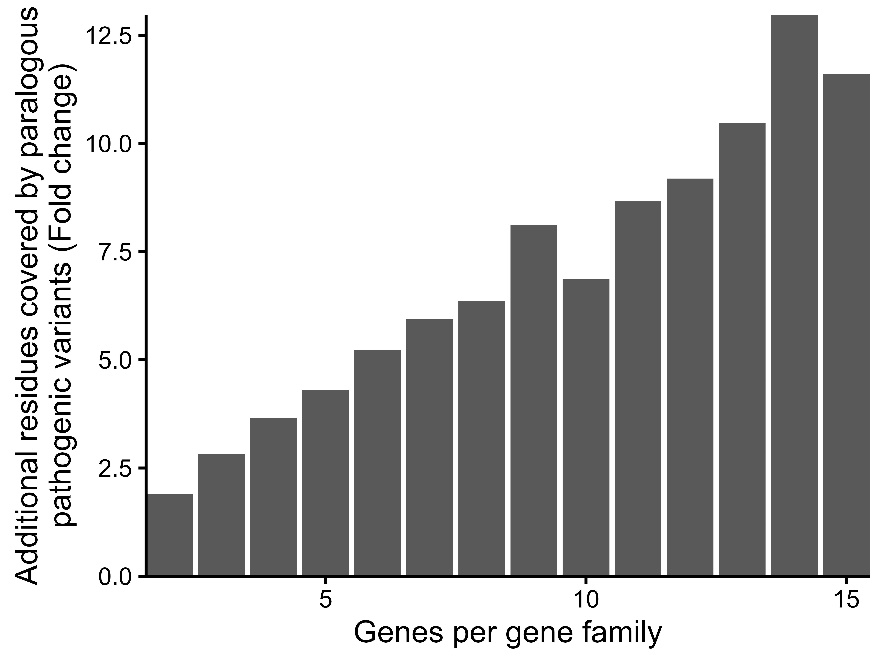


**Fig S1: The bar plot shows a fold-wise increase in pathogenic variant residue coverage with consideration of paralog genes compared to the same gene only.** The fold-change is stratified by the number of genes in a gene family.

**
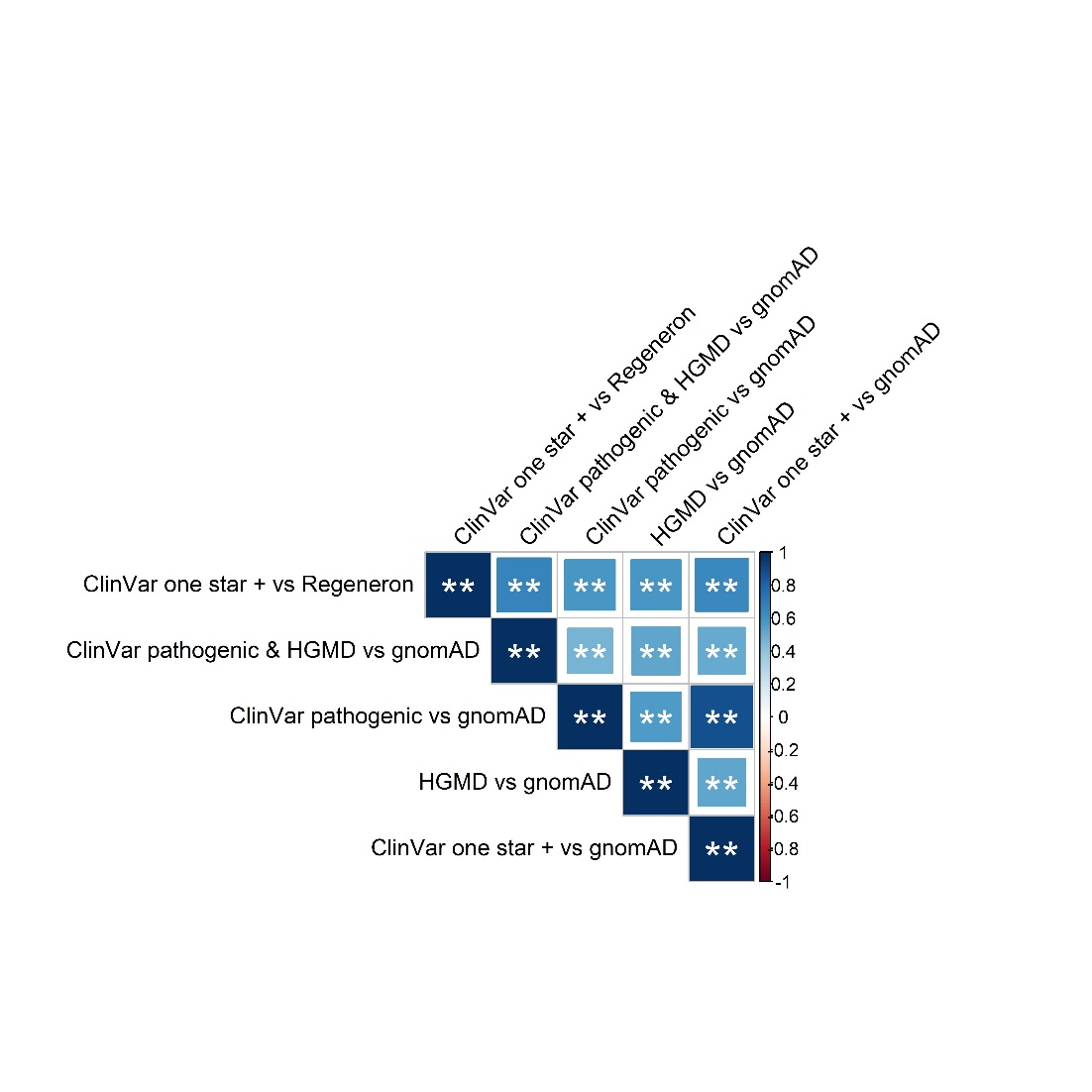
Fig S2: Pearson correlation matrix of LR+ values across five different dataset compositions.** Each cell shows the correlation between the LR+ estimates for pathogenic variants for the para-DIFF and para-SAME criterium: 1) ClinVar pathogenic variants (all review stars) plus HGMD vs gnomAD, 2) ClinVar pathogenic variants (all review stars) vs gnomAD, 3) HGMD variants vs gnomAD, 4) ClinVar pathogenic variants (≥1 review star) vs gnomAD, 5) ClinVar pathogenic variants (≥1 review star) vs a Regeneron exome dataset non-overlapping with gnomAD. High correlations in the heatmap indicate that the LR+ estimates remain consistent across all dataset comparisons. **: significant at p < 0.001 after Bonferroni multiple testing correction.


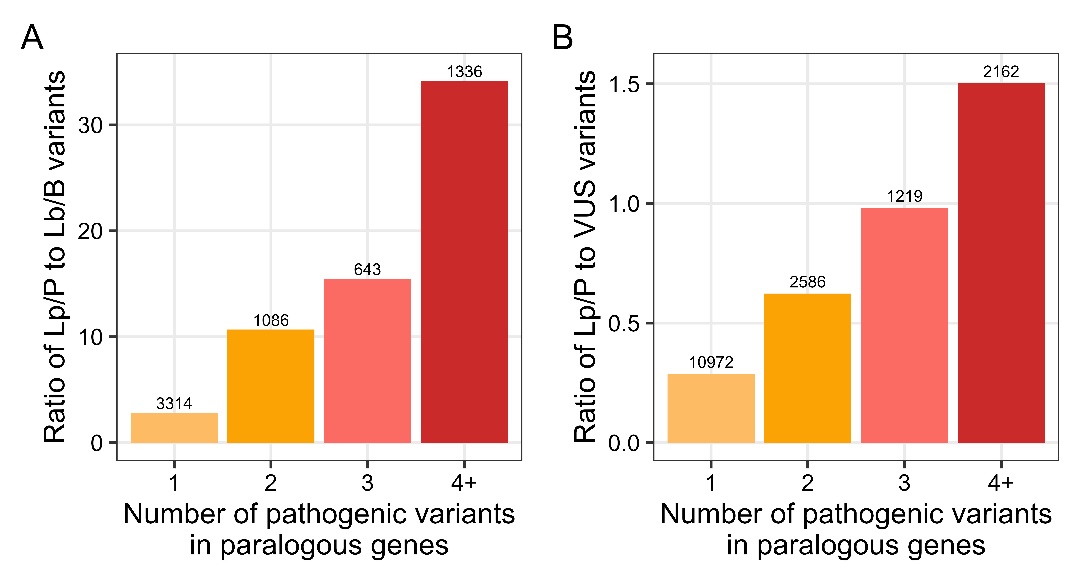


**Fig S3: The likelihood that a variant is pathogenic increases with the number of paralogous pathogenic variants at the same position.** The bar plots show the ratio of the number of likely pathogenic (LP) and pathogenic (P) variants relative to i) likely benign (LB) and benign (B) variants (left panel) and ii) variants of uncertain significance (VUS) (right panel). This ratio is computed separately for positions where 1, 2, 3, or 4+ paralogous pathogenic variants have been observed (x-axis). The total number of LP/P and LB/B variants (left panel) and of LP/P and VUS variants (right panel) in each category is displayed above the corresponding bars. A higher ratio indicates a greater enrichment of LP/P variants relative to LB/B or VUS variants at the same protein residue. Data were obtained from ClinVar (accessed December 2024).

**
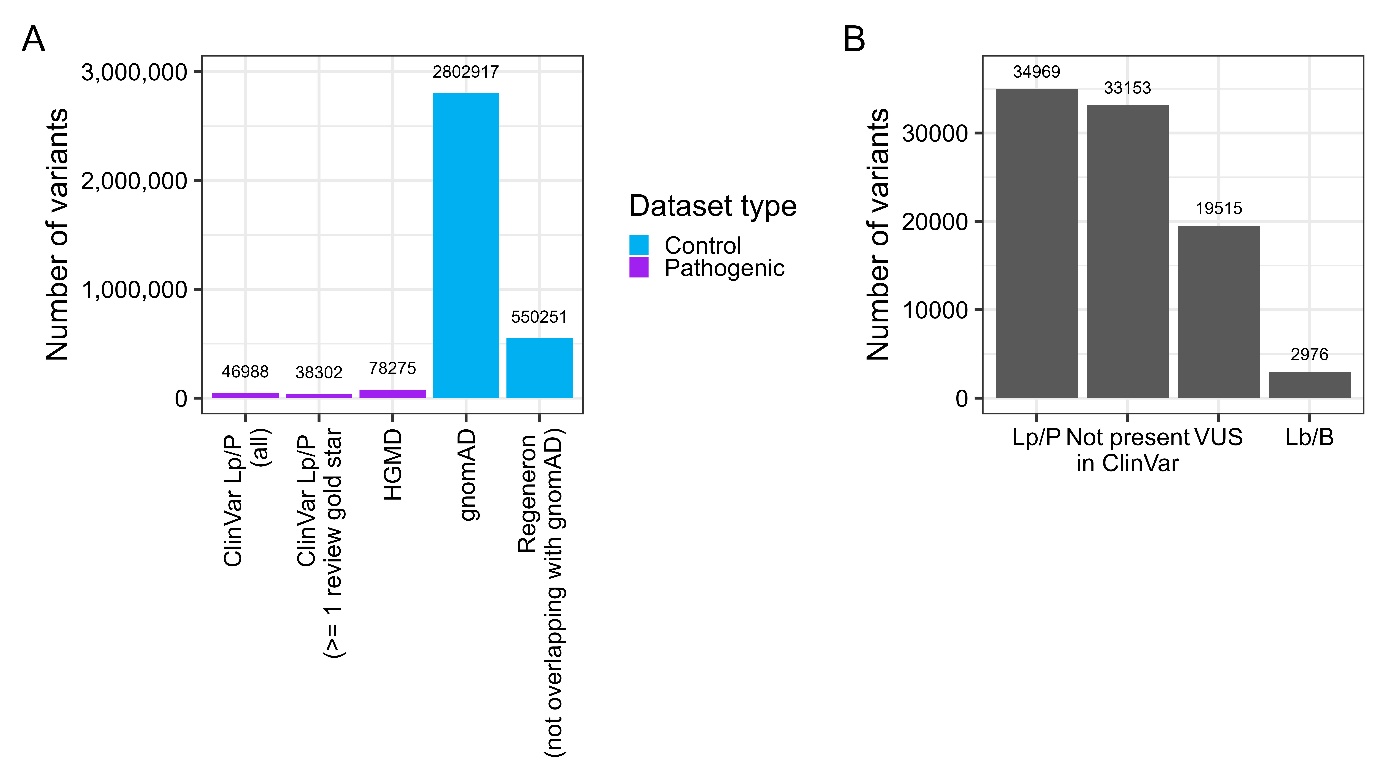
**

**Fig S4:** Overview of variant datasets and classification overlap. A) The number of variants included in the study from patient and population control datasets. Pathogenic variants were derived from ClinVar (all "Likely Pathogenic/Pathogenic" [Lp/P], see methods for details) and the Human Gene Mutation Database (HGMD, "Disease-causing" [DM] variants). Population control variants were sourced from gnomAD (v4.1.0) and the Regeneron Genetics Center One Million Exome Variant dataset (v.1.1.3, non-overlapping with gnomAD). B) Overlap of HGMD variants with ClinVar classifications. Variants are categorized as "Likely Pathogenic/Pathogenic" (Lp/P), "Not present in ClinVar," "Variant of Uncertain Significance" (VUS), and "Likely Benign/Benign" (Lb/B). The total number of variants for each category is shown above the bars.


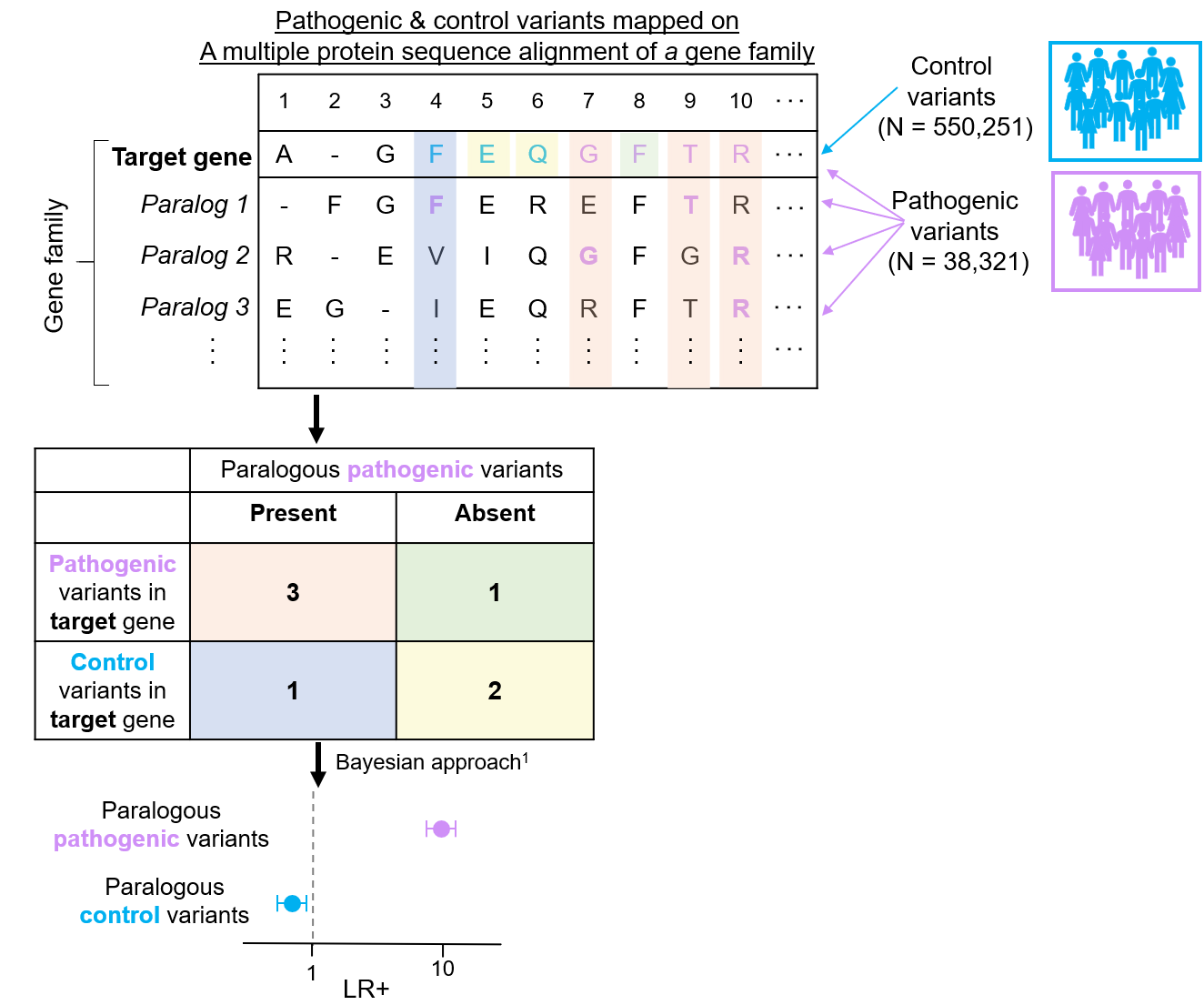


**Fig S5: Calculation of the likelihood ratio in the presence of pathogenic paralogous variants.** ^1^Approach applied to determine the strength of ACMG criteria in Tavtigian et al. 2018.
